# miR-1297 sensitizes glioma cells to temozolomide (TMZ) treatment through targeting adrenomedullin (ADM)

**DOI:** 10.1186/s12967-022-03647-6

**Published:** 2022-10-01

**Authors:** Zongze He, Meixiong Cheng, Junting Hu, Lingtong Liu, Ping Liu, Longyi Chen, Deqian Cao, Jian Tang

**Affiliations:** 1 Department of Neurosurgery, Sichuan Provincial People’s Hospital, University of Electronic Science and Technology of China, No.32 West Second Section First Ring Road, Chengdu, 610072 Sichuan China; 2 Chinese Academy of Sciences Sichuan Translational Medicine Research Hospital, Chengdu, 610072 China

**Keywords:** Gliomas, Temozolomide (TMZ), Sensitivity, Adrenomedullin (ADM), miR-1297

## Abstract

**Background:**

Gliomas account for about 80% of all malignant brain and other central nervous system (CNS) tumors. Temozolomide (TMZ) resistance represents a major treatment hurdle. Adrenomedullin (ADM) has been reported to induce glioblastoma cell growth.

**Methods:**

Cell viability was measured using the CCK-8 assay. The apoptosis analysis was performed using the Annexin V-FITC Apoptosis Detection Kit. The mitochondrial membrane potential was determined by JC-1 staining. A nude mouse tumor assay was used to detect tumor formation. Hematoxylin and eosin (H&E) and immunohistochemical (IHC) staining were performed in tissue sections. Activation of Akt and Erk and expression of apoptosis-related proteins were determined by immunoblotting.

**Results:**

ADM expression has been found upregulated in TMZ -resistant glioma samples based on bioinformatics and experimental analyses. Knocking down ADM in glioma cells enhanced the suppressive effects of TMZ on glioma cell viability, promotive effects on cell apoptosis, and inhibitory effects on mitochondrial membrane potential. Moreover, ADM knockdown also enhanced TMZ effects on Bax/Bcl-2, Akt phosphorylation, and Erk1/2 phosphorylation. Bioinformatics and experimental investigation indicated that miR-1297 directly targeted ADM and inhibited ADM expression. miR-1297 overexpression exerted similar effects to ADM knockdown on TMZ-treated glioma cells. More importantly, under TMZ treatment, inhibition of miR-1297 attenuated TMZ treatment on glioma cells; ADM knockdown partially attenuated the effects of miR-1297 inhibition on TMZ-treated glioma cells.

**Conclusions:**

miR-1297 sensitizes glioma cells to TMZ treatment through targeting ADM. The Bax/Bcl-2, Akt, and Erk1/2 signaling pathways, as well as mitochondrial functions might be involved.

**Supplementary Information:**

The online version contains supplementary material available at 10.1186/s12967-022-03647-6.

## Introduction

Gliomas, as common primary brain tumors, account for about 80% of all malignant brain and other central nervous system (CNS) tumors [1]. Glioblastoma is a grade IV glioma, which is the most aggressive form and accounts for 50% of all gliomas. Although progress has been made in treatment, glioblastoma patients still have a very bad prognosis, with only 14–17-month median survival [2]. The current standard treatment options include surgery, radiotherapy and chemotherapy, especially temozolomide (TMZ), which is the most commonly used clinical chemotherapy for Glioblastoma [3, 4]. TMZ can efficiently cross the blood–brain barrier and induce glioma cell apoptosis; however, chemotherapy resistance often develops and represents a major treatment hurdle. Since glioma resistance to TMZ is associated with multiple factors, identifying more factors that enhance glioma cell sensitivity to TMZ may be an effective intervention to improve the prognosis of patients.

Adrenomedullin (ADM), an effective vasodilator peptide composed of 52 amino acids, is first found in human pheochromocytoma. ADM shows expression in human lung, breast, brain, prostate, colon, and other tumor cell lines [5]. ADM mRNA expression in brain tumors is correlated with the type and grade of tumors [6]. In an oncology environment, ADM influences tumor cell growth, angiogenesis and apoptosis [5, 7, 8]. In gliomas, ADM expression levels have been found upregulated, and ADM acts as an effective inducer of the growth of glioblastoma cells [6]. As previously reported, ADM exerts a significant effect on multiple critical pathways, for example, cAMP, PI3K/Akt-dependent, and Erk signaling. Through the PI3K/Akt-dependent signaling, ADM causes endothelium-dependent vasorelaxation [9], and the infusion of ADM relieves myocardial ischemia or reperfusion injury [10]. In addition, through MAPK/Erk activation, ADM signaling modulates additional downstream pathways which enhance the growth and survival of endothelial cells [11]. ADM can also upregulate Bcl-2 to exert protective effects on cancer cells against hypoxia-induced apoptosis through autocrine or paracrine modes of action [12]. Notably, blocking PI3K/Akt/mTOR signaling has been considered to be a complementary target to further overcome the resistance of glioblastoma under hypoxic conditions [13]. Sato et al. [14] revealed that TMZ in combination with the MEK inhibitor SL327 acts synergistically to sensitize glioblastoma stem-like cells to TMZ treatment. Thus, agents that could inhibit ADM expression might sensitize glioma cells to TMZ treatment.

MicroRNAs (miRNAs) have been found to act as critical posttranscriptional gene expression ‘fine-tuners’ within normal and tumor progression [15–17]. miRNAs generally regulate gene expression via interacting with target mRNA 3ʹ-untranslated region (3ʹ-UTR) through mRNA destabilization and translation repression. Mounting evidence indicates that dysregulated miRNA expression signatures are tightly associated with various tumors, such as glioblastoma, and play context-dependent actions in tumorigenicity regulation and therapy response [16, 18–21]. Interestingly, several miRNAs, including miR-27a-3p [22], miR-93 [23], miR-26a [24], miR-1238 [25], miR-129-5p [26], miR-519a [27] and so on, have been reported involved in glioma resistance to TMZ treatment. Thus, searching for miRNAs that might target ADM might be a promising strategy for improving glioma resistance to TMZ.

Herein, we verified ADM expression in TMZ-resistant and -sensitive glioma samples using bioinformatics and experimental analyses. ADM knockdown was achieved in glioma cells and the specific effects of ADM knockdown on glioma sensitivity to TMZ were investigated. We employed five online tools to predict miRNAs targeting ADM; miR-1297 was selected after confirming the expression profiles of predicted candidates. The predicted miR-1297 binding to ADM and miR-1297 regulation of ADM were verified. The specific effects of miR-1297 overexpression on glioma sensitivity to TMZ were investigated. Lastly, the dynamic effects of the miR-1297/ADM axis on glioma sensitivity to TMZ were investigated.

## Materials and methods

### Clinical samples collection

A total of 18 glioma tissue samples and 18 noncancerous adjacent tissue samples (peritumoral brain edema tissue) were obtained from patients undergoing surgical treatment at Sichuan Provincial People’s Hospital under the approval of the Ethics Committee of Sichuan Provincial People’s Hospital. All enrolled patients provided written informed consent. All the tissues were kept at − 80 °C or fixed in 4% paraformaldehyde before further experiments.

### Bioinformatic analysis

Expression data from Gene Expression Omnibus (GEO) datasets, The Cancer Genome Atlas Glioma (TCGA-GBMLGG) were downloaded. GSE68029 contains the expression profiling of 6 untreated glioblastoma stem cells (GSC) and 6 resistant GSC clones (survived from 500 μM TMZ treatment). GSE113510 contains the expression profiling of 3 LN-229 cells and 3 TMZ-resistant LN-229 cells. GSE46531 contains the expression profiling of 6 radiation-treated GSCs and 6 radiation-treated TMZ-resistant GSCs. The expression of ADM in different groups was analyzed by Student’s t-test (P-value < 0.05). For overall survival analysis of ADM, the expression matrix of TCGA-GBMLGG (n = 625) and Chinese Glioma Genome Atlas (CGGA) (n = 601) and correlated survival data were analyzed by R language packages survival and surviminer.

### Cell treatment

Human non-cancerous glial cell line SVG p12 (CRL-8621) and human glioma cell lines [U87-MG (HTB-14) and T98G (CRL-1690)] were cultured (37 °C, 5% CO_2_) in Eagle's Minimum Essential Medium (EMEM; 30–2003) containing 10% FBS (Gibco, Waltham, MA, USA). Human glioma cell lines [A-172 (CRL-1620) and LN-229 (CRL-2611)] were kept (37 °C, 5% CO_2_ in 10% FBS (Gibco)-supplemented Dulbecco's Modified Eagle's Medium (DMEM; 30–2002). All cells and medium were obtained from ATCC (Manassas, VA, USA). For TMZ treatment, cells were exposed to 100 μM of TMZ (Sigma, USA) for 48 h and then collected for further investigation.

### Cell transfection

Short hairpin RNA targeting ADM (sh1-ADM or sh2-ADM) was used to achieve the knockdown of ADM (GenePharma, Shanghai, China). miR-1297 expression was intervened through the transfection of miR-1297 mimics/inhibitor (GenePharma). All the transfection was performed using Lipofectamine 3000 reagent (Invitrogen). Cells were seeded in 6/96-well plates. After transfection, cells were subjected to 24/48-h incubation for subsequent analysis.

### Cell counting kit-8 (CCK-8) for cell viability

Cell viability was measured using the CCK-8 (Beyotime, China). Cells (1.0 × 10^4^ cells/well) were seeded into 96-well plates. Following an overnight incubation (37 °C), cells underwent 4-h incubation (37 °C) with 10 μL CCK-8 in 100 μL serum-free DMEM. A microplate reader (Bio-Rad, model 680; Hercules, CA, USA) was utilized to determine the optical density at 490 nm.

### qRT-PCR

Total RNA of tissues and cells was extracted by TRIzol (Invitrogen, USA). miRNA and mRNA expressions were determined utilizing the SYBR Green PCR kit (Qiagen, Hilden, Germany). RNU6B (for miRNA) and GAPDH (for mRNA) served as endogenous references. Data were analyzed using the 2^−ΔΔCT^ method. The primer sequence was listed in Additional file 1: Table S1.

### Immunoblotting

Immunoblotting was used for measuring the protein levels of ADM, pro-caspase-3, pro-caspase-9, cleaved caspase-3/9, Bax, Bcl-2, cleaved PARP, Akt, p-Akt, Erk1/2, and p-Erk1/2. All cells were lysed in 1% PMSF-contained RIPA buffer. After extraction, protein samples were separated by loading onto SDS-PAGE gel, followed by transferring to PVDF membranes. After that, membranes were blocked with 2% bovine serum albumin in TBST, followed by an overnight incubation (4 °C) with primary antibodies: ADM (ab69117, Abcam, Cambridge, MA, USA), caspase-3 (19677-1-AP, Proteintech, Wuhan, China), caspase-9 (10380-1-AP, Proteintech), Bax (50599-2-Ig, Proteintech), Bcl-2 (12789-1-AP, Proteintech), cleaved PARP (13371-1-AP, Proteintech), Akt (Y409094, Applied Biological Materials Inc., Richmond, Canada), p-Akt (66444-1-1 g, Proteintech), Erk1/2 (67170-1-Ig, Proteintech), and p-Erk1/2 (sc-81492, Santa Cruz, Dallas, TX, USA). Next, membranes underwent an incubation with HRP-labeled secondary antibody, followed by visualization using ECL Substrates (Millipore, MA, USA). GAPDH served as an internal reference.

### Flow cytometry

The apoptosis analysis was performed using the Annexin V-FITC/PI Apoptosis Detection Kit (KeyGEN BioTech, Nanjing, China) following the aforementioned method [28]. The excitation (Ex) and emission (Em) wavelengths are 488 nm and 530 nm, separately. The FITC positive/PI positive and FITC positive/PI negative cells were considered as apoptotic cells. The negative control was shown in Additional file 2: Fig. S1. The mitochondrial membrane potential (ΔΨm) change was also determined by flow cytometry. Briefly, cells were collected and incubated with 10 µg/mL JC-1 staining solution (Yeasen, Shanghai, China) for 15 min at a cell culture incubator. Then, cells were analyzed by flow cytometry immediately (Ex = 488 nm, Em = 530 nm). The rate of Q2 quadrant represented no loss of ΔΨm.

### Dual-luciferase reporter assay

After ADM 3'-UTR amplification by PCR, wt-ADM 3'-UTR was constructed by cloning ADM 3'-UTR into the psiCHECK-2 vector (Promega, Madison, WI, USA) downstream of the Renilla gene. mut-ADM 3'-UTR was constructed by mutating ADM seed region for the removal of all complementarity to miR-1297. The above vectors were co-transfected with miR-1297 mimics/inhibitor into 293T cells. After transfection for 48 h, luciferase assays were carried out applying the Dual-Luciferase Reporter Assay System (Promega). The Renilla luciferase activities were normalized to firefly luciferase activities.

### Nude mice tumorigenicity assay

All animal protocols were performed under the approval of the Animal Care and Used Committee of Sichuan Provincial People’s Hospital. LN-229 cells were randomly divided into 4 groups and transfected with ADM stable knockdown vector (lentivirus mediated shRNA-ADM) and shRNA control vector (lentivirus mediated shRNA-NC) were harvested at logarithmic phase and resuspended into serum-free medium at 1 × 10^7^ per milliliter. Then, 0.2 mL cell suspension were subcutaneously injected into 4 weeks BALB/c nude mice (weighting at 18–20 g, purchased from Laboratory Animal Research Institute of Sichuan Provincial People’s Hospital), at the right side of armpit. When all the groups formed the tumor at approximately one week, mice were injected intraperitoneally with TMZ or DMSO (30 mg/kg/day) every 3 days for another 3 weeks [29]. At end of the experiment, the length (mm), width (mm) and height (mm) of tumor tissues were measured by a vernier caliper, and tumor volume was calculated according to the following formula: Volume = long × width^2^/2. All implanted mice were sacrificed and the tumor tissues were collected for the subsequent analysis.

### Histological analyses

Hematoxylin and eosin (H&E) and immunohistochemical (IHC) staining were performed as previously described [30]. Mice tumor samples were fixed in 4% paraformaldehyde in PBS overnight, paraffin-embedded, and cut into 5-μm-thick sections. IHC analysis was performed for the protein level and distribution of Ki-67 using anti-Ki-67 (ab15580; Abcam).

### Statistical analysis

Experimental data were represented as means ± standard deviation (SD). Differences among groups were identified using one-way analysis of variance (ANOVA), followed by the Tukey’s test. The GraphPad Prism software (ver. 7.0; GraphPad, La Jolla, CA, USA) was utilized for all statistical analyses. *P* < 0.05 was considered as statistically significant.

## Results

### ADM is specifically upregulated in glioma tissues and TMZ-resistant glioma cell strains

**Fig. 1:**
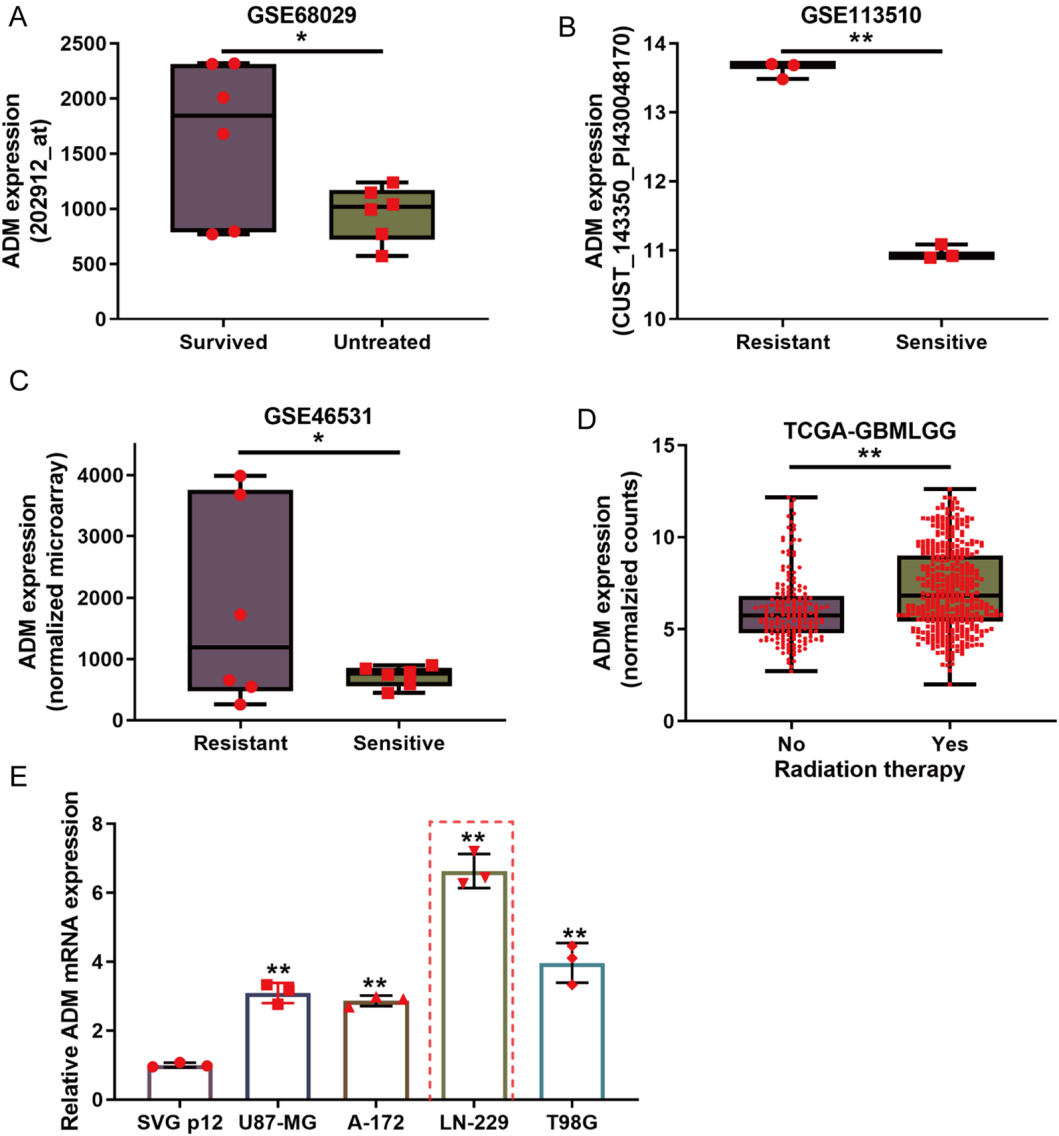
ADM is specifically up-regulated in TMZ-resistant glioblastoma cell strains. **A** The expression of ADM in untreated glioblastoma stem cells (GSCs) and GSCs that survived after 500uM temozolomide (TMZ) treatment, according to GSE68029. **B** The expression of ADM in induced TMZ-resistant LN229 cells and regular LN229 cells, according to GSE113510. **C** The expression of ADM in TMZ treatment sensitive glioblastoma stem cell clones and TMZ treatment resistant glioblastoma stem cell clones according to GSE46531. **D** The expression of ADM in samples with or without radiation-therapy, according to TCGA-GBMLGG. **E** ADM expression was determined in a normal human glial cell line SVG p12 and four glioma cell lines, U87-MG, A-172, LN-229, and T98G using qRT-PCR

For verifying ADM expression in TMZ-resistant glioma cells, several datasets downloaded from GEO were analyzed. According to GSE68029, ADM expression was significantly upregulated in glioblastoma stem cells (GSCs) that survived after 500 μM TMZ treatment compared with untreated GSCs (Fig. 1A). According to GSE113510, ADM expression was significantly upregulated in induced LN-229 cell lines with resistance to TMZ than that in regular LN-229 cell lines (Fig. 1B). According to GSE46531, ADM expression was significantly upregulated in TMZ-resistant glioblastoma stem cell clones (Fig. 1C). According to TCGA-GBMLGG, ADM expression was significantly upregulated samples with radiation-therapy compared with that in samples without radiation-therapy (Fig. 1D). In addition, according to GSE4412 and GSE4290, ADM levels were higher in the high-grade glioma tissue samples (Additional file 3: Fig. S2A-B). Moreover, the high expressed ADM was associated with poor overall survival of glioma patients based on CGGA and TCGA-GBMLGG datasets (Additional file 3: Fig. S2C and D). In the clinical collected samples, we also found that ADM mRNA and protein level was higher within advanced-stage samples compared with early-stage samples and adjacent non-cancerous peritumoral edema samples (Additional file 3: Fig. S2E and F). Then, ADM expression was determined within a normal human glial cell line SVG p12 and glioma cell lines (U87-MG, A-172, LN-229, and T98G) using qRT-PCR; ADM expression was dramatically increased within glioma cell lines than that within SVG p12 cell line, in particular in LN-229 cells (Fig. 1E).

### ADM knockdown sensitizes glioma cells to TMZ treatment

**Fig. 2:**
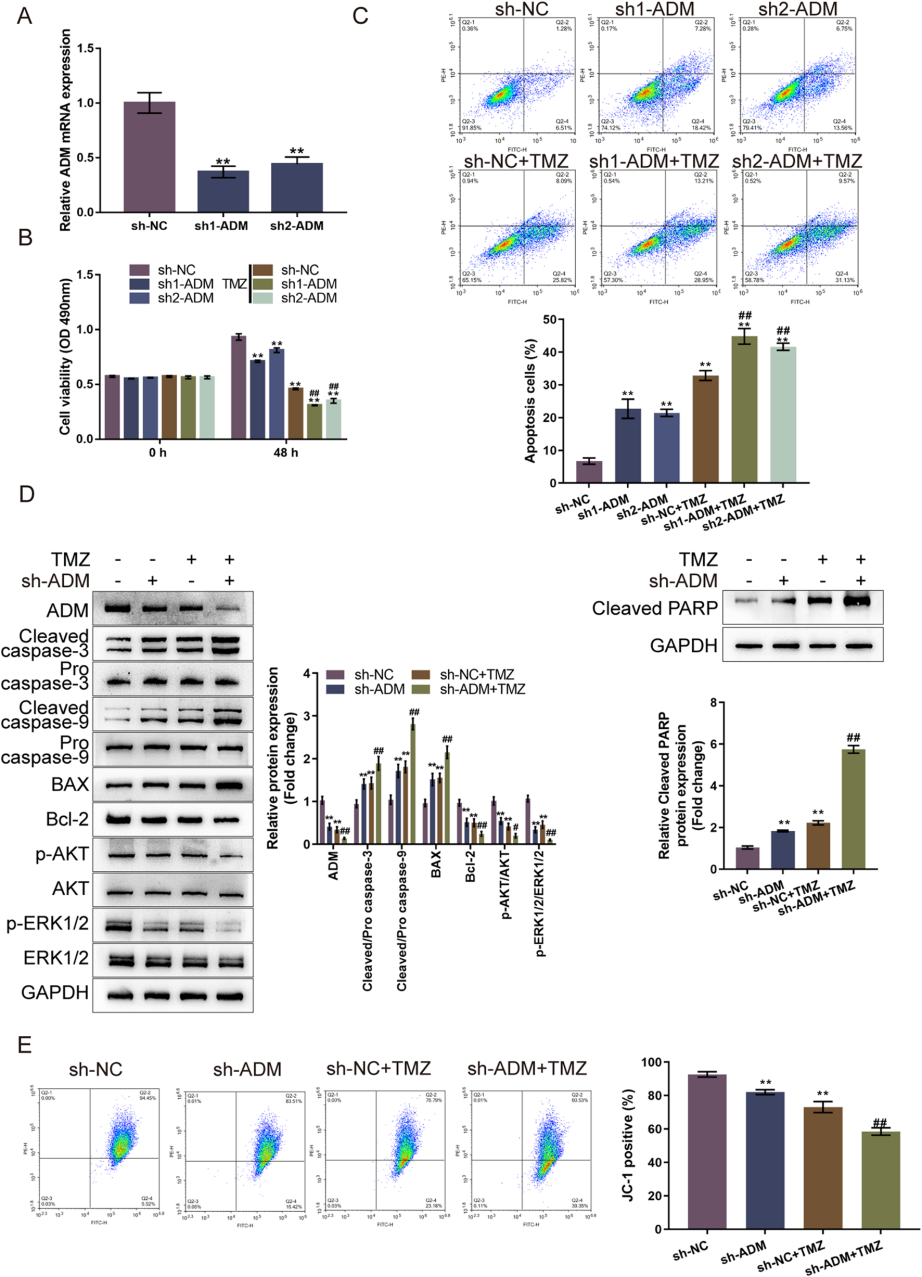
ADM knockdown sensitizes glioma cells to TMZ treatment. **A** ADM knockdown was achieved in LN-229 cells by transfecting short hairpin RNA targeting ADM (sh1-ADM or sh2-ADM); ADM knockdown was confirmed using qRT-PCR. Then, LN-229 cells were transfected with sh-NC or sh-ADM, treated or non-treated with TMZ, and examined for cell viability by CCK-8 assay (**B**); cell apoptosis by Flow cytometry (**C**); the protein levels of ADM, cleaved caspase 3, pro-caspase 3, cleaved caspase 9, pro-caspase 9, Bax, Bcl-2, p-Akt, Akt, p-Erk1/2, Erk1/2 and cleaved PARP by Immunoblotting (**D**); mitochondrial membrane potential changes by JC-1 staining (**E**)

Considering that ADM expression is upregulated in glioma tissues and TMZ-resistant glioma cells, ADM knockdown was achieved in LN-229 cells by transfecting short hairpin RNA targeting ADM (sh1-ADM or sh2-ADM); ADM knockdown was confirmed using qRT-PCR (Fig. 2A). Then, we transfected LN-229 cells with sh-NC or sh-ADM, treated or non-treated with TMZ, and examined for cellular phenotypes. As for cell viability, single sh1-ADM/sh2-ADM transfection or single TMZ treatment significantly suppressed cell viability compared with sh-NC or non-treatment group; ADM knockdown promoted TMZ inhibition upon the viability of glioma cells (Fig. 2B). As for cell apoptosis, single sh1-ADM/sh2-ADM transfection or single TMZ treatment significantly promoted cell apoptosis compared with sh-NC or non-treatment group, which was enhanced by ADM knockdown (Fig. 2C). Moreover, the sh1-ADM showed a better effect. Therefore, it was selected for further investigation. Single sh-ADM transfection or single TMZ treatment significantly decreased the protein levels of ADM, Bcl-2, p-Akt/Akt, and p-Erk1/2/Erk1/2, along with the elevated cleaved caspase 3/9, Bax and cleaved PARP levels; ADM knockdown enhanced TMZ effects on these factors (Fig. 2D). Moreover, ΔΨm could be diminished by ADM transfection or TMZ treatment, and ADM knockdown further enhanced the suppressive effects of TMZ on ΔΨm (Fig. 2E).

### In vivo effects of ADM knockdown on the TMZ treatment of glioma

**Fig. 3:**
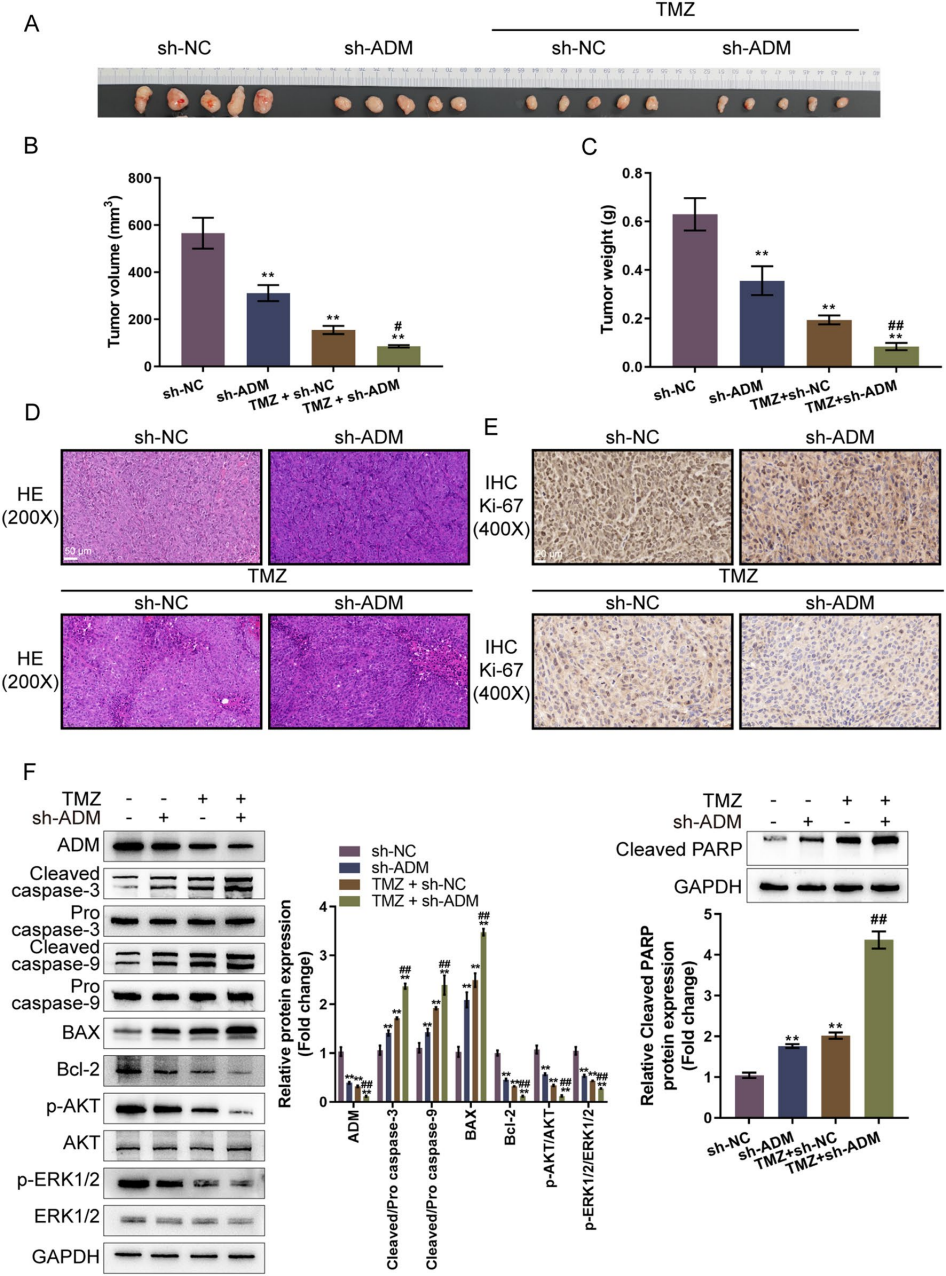
In vivo effects of ADM knockdown on the TMZ treatment of glioma **A** A subcutaneous tumor model was established in nude mice as described, and part of the model mice received TMZ treatment or ADM knockdown and the images of the tumors were displayed. **B**, **C** Tumor weight and volume were examined. **D** The histopathological characteristics of the tumors were examined by hematoxylin and eosin (H&E) staining. **E** The level of proliferation marker protein Ki-67 was detected in the tumors by immunohistochemistry (IHC) staining. **F** The protein levels of ADM, cleaved caspase 3, pro caspase 3, cleaved caspase 9, pro-caspase 9, Bax, Bcl-2, p-Akt, Akt, p-Erk1/2, Erk1/2 and cleaved PARP in the tumors were determined by Immunoblotting

To further explore the effects of ADM knockdown on the TMZ treatment of glioma cells in vivo, a subcutaneous tumor model in nude mice was established and part of the mouse model received TMZ treatment. Either single ADM knockdown or TMZ treatment significantly decreased tumor sizes, volume and weight; ADM knockdown enhanced the efficacies of TMZ in glioma (Fig. 3A–C). H&E staining was then performed to examine the histopathological characteristics of the tumors; Fig. 3D showed that ADM knockdown or TMZ treatment increased the necrosis of tumor tissues. Then, the level of proliferation marker protein Ki-67 was detected in the tumors by IHC staining. Either single ADM knockdown or TMZ treatment notably decreased Ki-67 protein; ADM knockdown enhanced the effect of TMZ in glioma (Fig. 3E). Single ADM knockdown or single TMZ treatment significantly decreased the protein levels of ADM, Bcl-2, p-Akt/Akt, and p-Erk1/2/Erk1/2, while elevated the cleaved caspase 3/9, Bax and cleaved PARP levels in tumors; and ADM knockdown enhanced TMZ effects on these factors (Fig. 3F).

### miR-1297 is an upstream regulatory miRNA for ADM

**Fig. 4:**
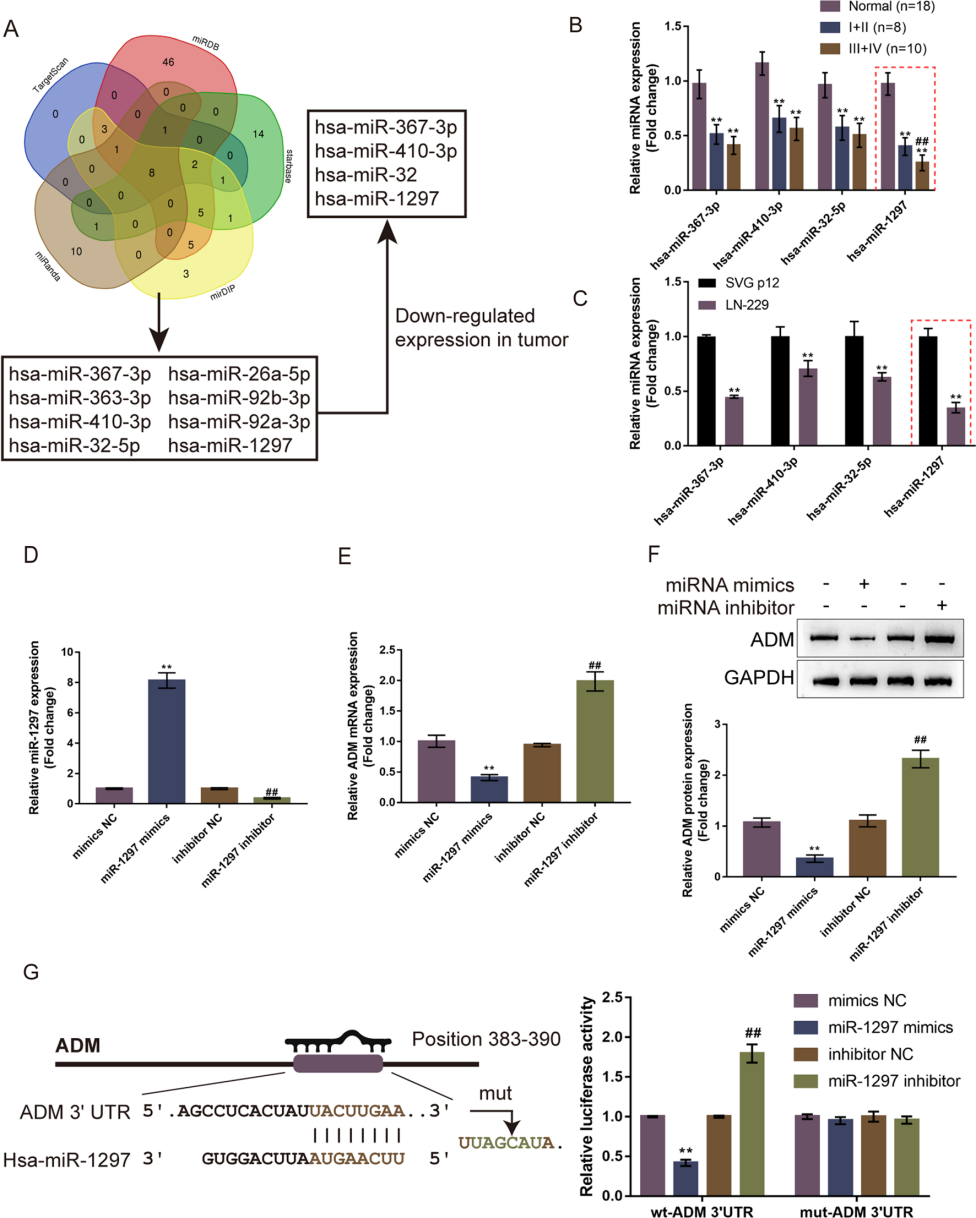
miR-1297 is an upstream regulatory miRNA for ADM **A** TargetScan, miRDB, miRanda, mirDIP, and Starbase were used to predict miRNAs that might target ADM; cross-checked results were compared with miRNAs downregulated in tumor samples. **B** The expression levels of four candidates were examined in normal non-cancerous samples (n = 18), stage I + II glioma samples (n = 8), and stage III + IV glioma samples (n = 10) using qRT-PCR. **C** The expression levels of four candidates were examined in a normal human glial cell line SVG p12 and a glioma cell line LN-229 using qRT-PCR. **D** miR-1297 overexpression or inhibition was achieved in LN-229 cells by transfecting miR-1297 mimics or inhibitor; miR-1297 overexpression or inhibition was confirmed using qRT-PCR. **E**, **F** LN-229 cells were transfected with miR-1297 mimics or inhibitor and examined for ADM expression using qRT-PCR and ADM protein levels by Immunoblotting. **G** Wild- and mutant-type ADM luciferase reporters were constructed as described and co-transfected in 293 T cells with miR-1297 mimics or inhibitor; luciferase activity was determined

Considering the crucial role of miRNAs in glioma chemo-resistance [31, 32], next, miRNAs that might target ADM were analyzed. miRNAs that may target ADM were predicted using TargetScan, miRDB, miRanda, mirDIP, and Starbase; 8 miRNAs (hsa-miR-367-3p, hsa-miR-26a-5p, hsa-miR-363-3p, hsa-miR-92b-3p, hsa-miR-410-3p, hsa-miR-92a-3p, hsa-miR-32-5p, and hsa-miR-1297) were identified (Fig. 4A). Among these 8 miRNAs, hsa-miR-367-3p [33, 34], hsa-miR-410-3p [35], hsa-miR-32-5p [36, 37], and hsa-miR-1297 [38–41] have been reported downregulated in gliomas before (Fig. 4A). We examined the expression of these four miRNAs within normal non-cancerous samples (n = 18), and glioma samples (8 stage I + II and 10 stage III + IV) using qRT-PCR; Fig. 4B showed that although four miRNAs were decreased within glioma samples, hsa-miR-1297 was dramatically decreased within advanced-stage samples compared with early-stage samples. Consistently, these four miRNAs were decreased in LN-229 cells relative to those in SVG p12 cells (Fig. 4C). Thus, we chose miR-1297 for subsequent analysis.

miR-1297 mimics/inhibitor was transfected to LN-229 cells for miR-1297 overexpression/inhibition, as confirmed by qRT-PCR (Fig. 4D). In LN-229 cells, miR-1297 overexpression downregulated ADM expression and decreased ADM protein level, while miR-1297 inhibition upregulated ADM expression and increased ADM protein level (Fig. 4E, F). Then, we constructed wild- and mutant-type ADM luciferase reporters, which were then co-transfected with miR-1297 mimics/inhibitor into 293 T cells, followed by the examination of luciferase activity. As shown by Fig. 4G, when co-transfected with wt-ADM and miR-1297 mimics/inhibitor, miR-1297 overexpression suppressed, whereas miR-1297 inhibition enhanced wt-ADM luciferase activity. However, co-transfection of mut-ADM with miR-1297 mimics/inhibitor failed to change the luciferase activity of mut-ADM.

### miR-1297 overexpression sensitizes glioma cells to TMZ

**Fig. 5:**
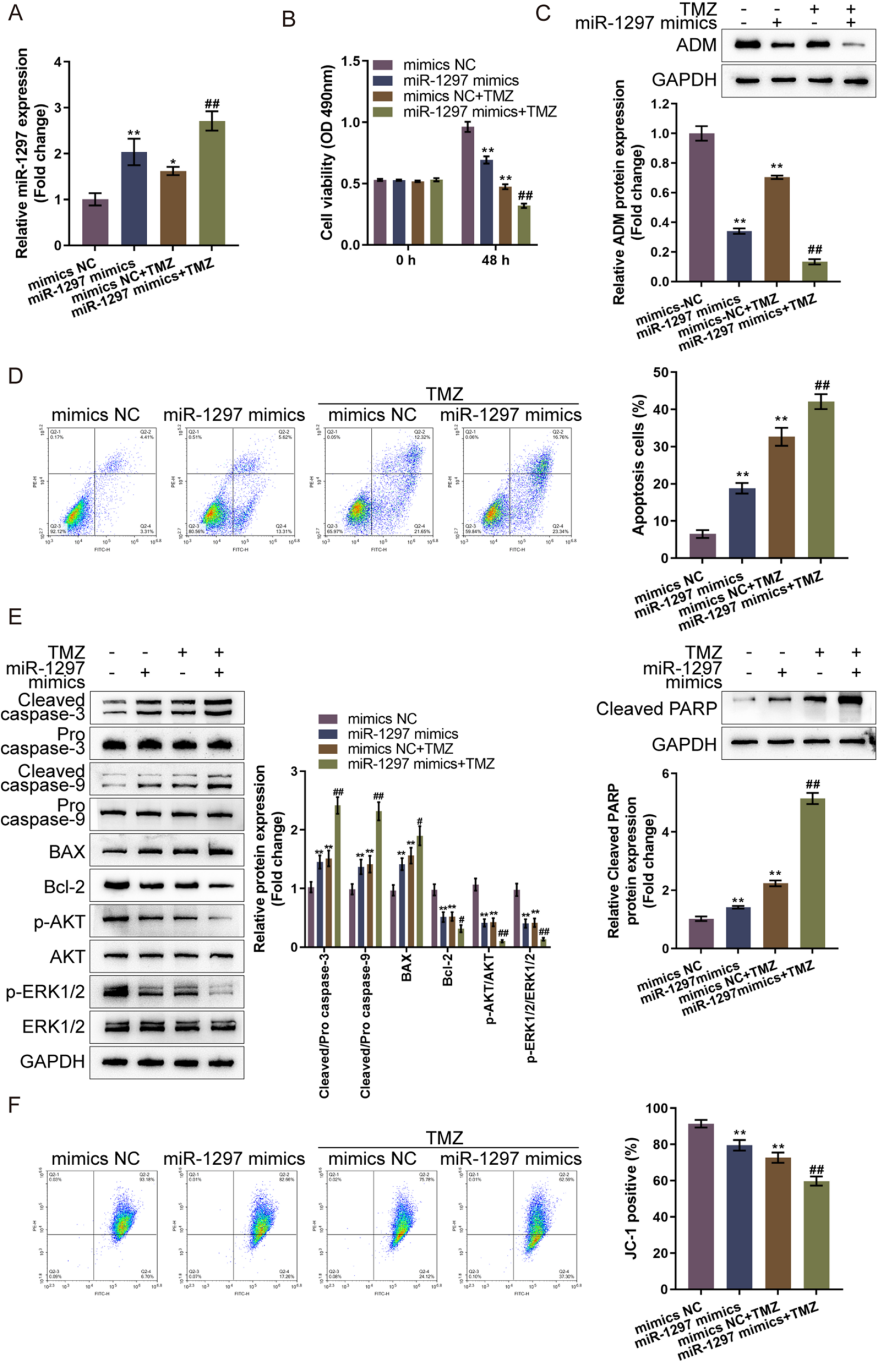
miR-1297 overexpression sensitizes glioma cells to TMZ treatment LN-229 cells were transfected with mimics NC or miR-1297 mimics, treated or non-treated with TMZ, and examined for miR-1297 expression by RT-PCR (**A**); cell viability by CCK-8 assay (**B**); ADM expression by immunoblotting (**C**); cell apoptosis by Flow cytometry (**D**); the protein levels of cleaved caspase 3, pro-caspase 3, cleaved caspase 9, pro-caspase 9, Bax, Bcl-2, p-Akt, Akt, p-Erk1/2, and Erk1/2 by Immunoblotting (**E**); mitochondrial membrane potential changes by JC-1 staining (**F**)

Considering that knockdown of ADM sensitizes glioma cells to TMZ and that miR-1297 targets ADM to inhibit ADM expression, we investigated the specific effects of miR-1297 upon TMZ sensitivity of glioma cells. We transfected LN-229 cells with mimics NC/miR-1297 mimics, treated or non-treated these cells with TMZ, and examined for cell phenotypes. Firstly, the level of miR-1297 was determined. miR-1297 levels were effectively increased by miR-1297 mimics and TMZ treatment (Fig. 5A). In contrast, miR-1297 mimics reduced ADM expression and further enhanced the reduction effect of TMZ on ADM expression (Fig. 5C). miR-1297 overexpression alone inhibited the viability of glioma cells and enhanced TMZ inhibition upon the viability of glioma cells (Fig. 5B). Consistently, miR-1297 overexpression alone promoted cell apoptosis and enhanced TMZ promotion upon glioma cell apoptosis (Fig. 5D). miR-1297 overexpression also notably reduced the levels of Bcl-2, p-Akt/Akt, and p-Erk1/2/Erk1/2, together with the increased cleaved caspase 3/9, Bax and cleaved PARP levels; overexpressed miR-1297 enhanced TMZ effects on these factors (Fig. 5E). Moreover, ΔΨm was also diminished by miR-1297 overexpression, and miR-1297 overexpression enhanced the suppressive effects of TMZ on ΔΨm (Fig. 5F). Thus, miR-1297 also enhances glioma cell sensitivity to TMZ treatment.

### ADM knockdown partially attenuated the effects of miR-1297 inhibition on TMZ-treated glioma cells

**Fig. 6:**
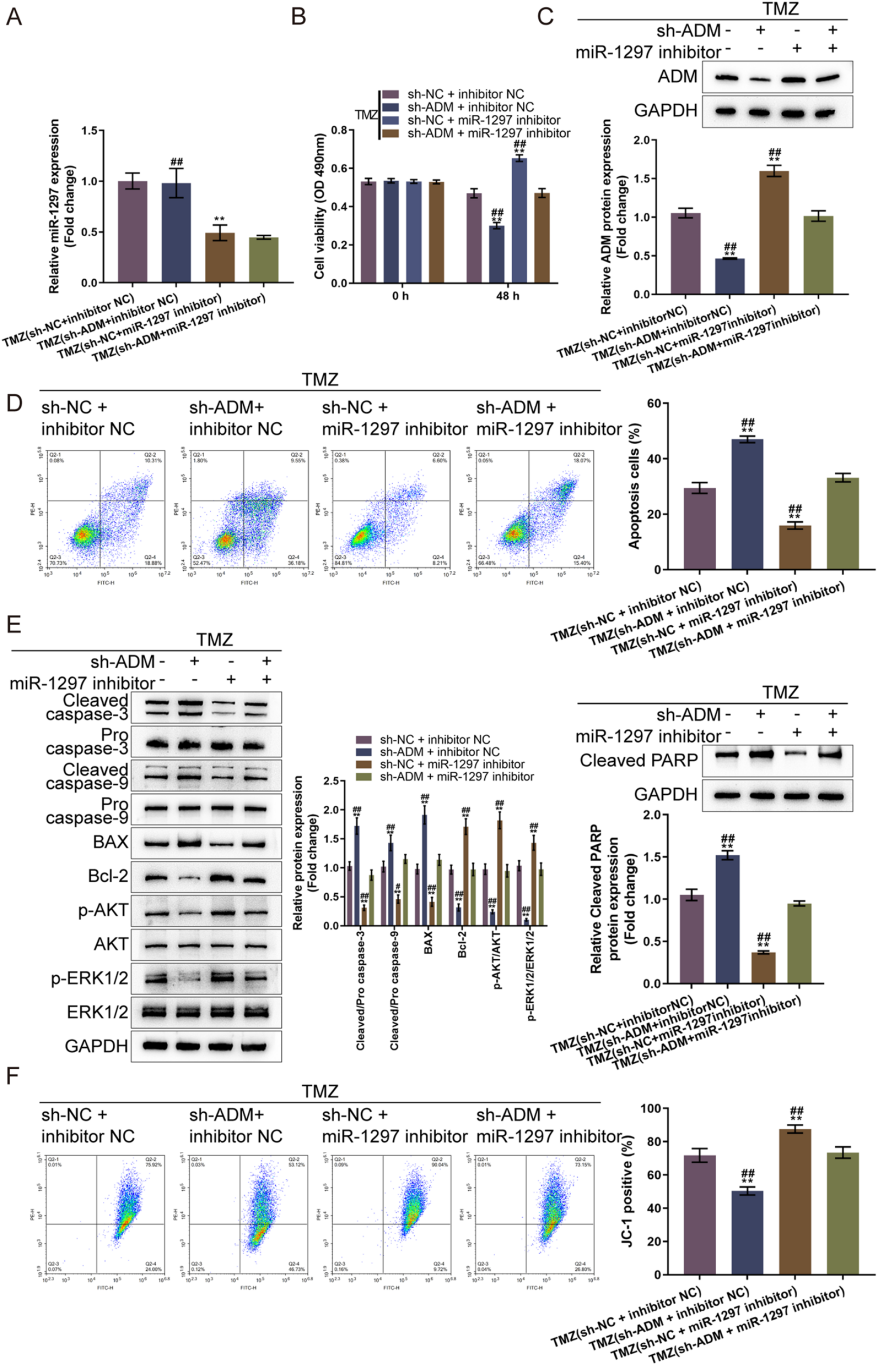
ADM knockdown partially attenuated the effects of miR-1297 inhibition on TMZ-treated glioma cells LN-229 cells were co-transfected with sh-ADM and miR-1297 inhibitor, treated with TMZ, and examined for miR-1297 expression by RT-PCR (**A**); cell viability by CCK-8 assay (**B**); ADM expression by immunoblotting (**C**); cell apoptosis by Flow cytometry (**D**); the protein levels of cleaved caspase 3, pro-caspase 3, cleaved caspase 9, pro-caspase 9, Bax, Bcl-2, p-Akt, Akt, p-Erk1/2, and Erk1/2 by Immunoblotting (**E**); mitochondrial membrane potential changes by JC-1 staining (**F**)

Lastly, since miR-1297 targets ADM to inhibit ADM expression, it was investigated that whether miR-1297/ADM axis had a synergistic effect on glioma cell sensitivity to TMZ treatment. We co-transfected LN-229 cells with sh-ADM and miR-1297 inhibitor, treated with TMZ, and examined for miR-1297 and ADM expression and cell phenotypes. Under TMZ treatment, miR-1297 levels were effectively reduced by miR-1297 inhibitor and were not changed in response to ADM knockdown (Fig. 6A). While, miR-1297 inhibitor increased ADM expression which could be reversed by ADM knockdown (Fig. 3C). ADM knockdown inhibited, whereas miR-1297 inhibition promoted glioma cell viability; ADM knockdown partially attenuated miR-1297 inhibition effects on cell viability (Fig. 6B). Similarly, under TMZ treatment, ADM knockdown promoted, whereas miR-1297 inhibition suppressed glioma cell apoptosis; ADM knockdown partially attenuated miR-1297 inhibition effects on glioma cell apoptosis (Fig. 6D). Consistently, ADM knockdown decreased the protein levels of ADM, Bcl-2, p-Akt/Akt, and p-Erk1/2/Erk1/2, along with elevated cleaved caspase 3/9, Bax and cleaved PARP, while miR-1297 inhibition showed opposite effects; ADM knockdown partially attenuated miR-1297 inhibition effects on these proteins (Fig. 6E). ΔΨm was diminished by ADM knockdown but amplified by miR-1297 inhibition, and ADM knockdown partially attenuated miR-1297 inhibition effects on ΔΨm (Fig. 6F).

## Discussion

In the present study, ADM expression has been found upregulated in glioma tissues and TMZ- resistant glioma cells based on bioinformatics and experimental analyses. Knocking down ADM in glioma cells enhanced the suppressive effects of TMZ on glioma cell viability, promotive effects on cell apoptosis, and inhibitory effects on mitochondrial membrane potential. Moreover, ADM knockdown also enhanced TMZ effects on Bax/Bcl-2, Akt phosphorylation, and Erk1/2 phosphorylation. miR-1297 directly targeted ADM and inhibited ADM expression. miR-1297 overexpression exerted similar effects to ADM knockdown on TMZ-treated glioma cells. More importantly, under TMZ treatment, inhibition of miR-1297 attenuated TMZ treatment on glioma cells; ADM knockdown partially attenuated the effects of miR-1297 inhibition on TMZ-treated glioma cells.

ADM, a member of the calcitonin-family of peptides, has approximately 24% homology with calcitonin gene-related peptide (CGRP) [42]. Peptides of the CGRP family are extensively found in the body and exert crucial biological effects, such as mediating calcium regulation [43], glucose metabolism [44], and cardiovascular functions [45]. ADM has been reported as one of 5 candidate genes associated with glioma resistance to TMZ, which showed to be consistently linked to higher expression of DDIT4 and lower prognosis in TMZ-treated cells [46]. Adrenomedullin 2, another CGRP member, increased glioma cell invasion and proliferation via enhancing the formation of filopodia, which depends on the activation of Erk1/2 pathway [47]. In this study, ADM expression has been found upregulated in TMZ -resistant glioma samples, suggesting the underlying effect of ADM on glioma cell TMZ resistance. As expected, ADM knockdown in glioma cells amplified the suppression of TMZ on glioma cell viability and promotive effects on cell apoptosis. Moreover, ADM knockdown also enhanced TMZ effects on Bax/Bcl-2, Akt phosphorylation, and Erk1/2 phosphorylation. These data suggest that ADM knockdown might sensitize glioma cells to TMZ treatment, and the Bax/Bcl-2, Akt, and Erk1/2 signaling might be involved. Notably, ADM knockdown also enhanced the inhibitory effects of TMZ on mitochondrial membrane potential. Reportedly, ADM upregulated the mRNA expression and peptide level of oncogene Bcl-2 within Ishikawa cells under the condition of normoxia and anoxia. It has shown that Bcl-2 has protective effects in response to many different apoptotic stimuli, such as hypoxia [48]. Bcl-2 prevents apoptosis, which may be achieved by blocking the release of Cytochrome c from mitochondria, and then inhibiting caspase-3vactivation and the subsequent apoptotic effects [49]. Herein, ADM knockdown or TMZ treatment induced caspase 3 and caspase 9 cleavage, as well as mitochondrial membrane potential impairment, suggesting that mitochondrial function might also be involved in ADM knockdown enhancing glioma cell sensitivity to TMZ treatment.

As promising agents that could inhibit the expression of downstream target mRNAs, miRNAs play a critical role in carcinogenesis through regulating different targets’ expressions. Various studies have indicated that miRNAs can be efficient biomarkers of glioma and used as therapeutic targets/agents [50–52]. In this study, miR-1297 has been predicted as an upstream regulatory miRNA for ADM. Through direct targeting, miR-1297 inhibited ADM expression. It has been validated that miR-1297 exerts antitumor effects on glioma. Through regulating HMGA1, miR-1297 inhibited glioma cell growth in vivo and in vitro [39]. Another group demonstrated that miR-1297 inhibited in vitro glioma cell invasion, migration and proliferation through targeting EZH2 [38]. In this study, single miR-1297 overexpression enhanced TMZ effects on glioma cells, whereas miR-1297 inhibition attenuated TMZ effects; more importantly, ADM knockdown significantly attenuated miR-1297 inhibition effects on TMZ-treated glioma cells. These data indicate that miR-1297/ADM axis affects glioma cell sensitivity to TMZ treatment.

In conclusion, miR-1297 sensitizes glioma cells to TMZ treatment through targeting ADM. The Bax/Bcl-2, Akt, and Erk1/2 signaling pathways, as well as mitochondrial functions might be involved.

## Supplementary Information

**Additional file 1: Table S1**. The primer sequence.

**Additional file 2: Fig. S1**. The negative control of flow cytometry analysis.

**Additional file 3: Fig. S2**. ADM was upregulated in glioma tissues based on online datasets and clinical collected samples. (A) GSE4412; (B) GSE4290; (C) overall survival of ADM according to CGGA dataset; (D) overall survival of ADM according to TCGA-GBMLGG dataset; (E and F) The mRNA and protein levels of ADM in glioma samples and non-cancerous peritumoral brain edema tissue.

## Data Availability

Please contact the authors for data requests.
