# Supplementary material for: miR-1297 sensitizes glioma cells to temozolomide (TMZ) treatment through targeting adrenomedullin (ADM)
Source: J Transl Med. 2022 Oct 1;20:443. doi: 10.1186/s12967-022-03647-6 (PMC9526964; doi:10.1186/s12967-022-03647-6)
Supplement: Supplementary file 1 — Additional file 1: Table S1. The primer sequence. [file 12967_2022_3647_MOESM1_ESM.docx]

Table S1 the primer sequence

| RT-PCR | Forward | Reverse |
| --- | --- | --- |
| ADM | ATGAAGCTGGTTTCCGTCG | GACATCCGCAGTTCCCTCTT |
| GAPDH | ACAGCCTCAAGATCATCAGC | GGTCATGAGTCCTTCCACGAT |
| MiR-367-3p | RT：GTCGTATCCAGTGCGTGTCGTGGAGTCGGCAATTGCACTGGATACGACTCACCA  F：GCCAATTGCACTTTAGCAA | GCCGCTGGTGACATG |
| MiR-410-3p | RT：GTCGTATCCAGTGCGTGTCGTGGAGTCGGCAATTGCACTGGATACGACACAGGC  F:GCCGCAATATAACACAGATG | GCCGCTGGTGACATG |
| MiR-32-5p | RT：GTCGTATCCAGTGCGTGTCGTGGAGTCGGCAATTGCACTGGATACGACTGCAAC  F:GCCGGTATTGCACATTACTAA | GCCGCTGGTGACATG |
| MiR-1297 | RT：GTCGTATCCAGTGCGTGTCGTGGAGTCGGCAATTGCACTGGATACGACCACCTG  F:GCCGCCGTTCAAGTAATT | GCCGCTGGTGACATG |
| U6 | CTCGCTTCGGCAGCACA | AACGCTTCACGAATTTGCGT |
| Sh1-ADM | GATCCGCAAGCCTCACTATTACTTGACTCGAGTCAAGTAATAGTGAGGCTTGCTTTTTG | AATTCAAAAAGCAAGCCTCACTATTACTTGACTCGAGTCAAGTAATAGTGAGGCTTGCG |
| Sh2-ADM | GATCCGCAATGCGTGTTGTACATACACTCGAGTGTATGTACAACACGCATTGCTTTTTG- | AATTCAAAAAGCAATGCGTGTTGTACATACACTCGAGTGTATGTACAACACGCATTGCG |
| Sh-NC | GATCCGCAGATGAAGGCACGGTCACGCTCGAGGCAGATGAAGGCACGGTCACGTTTTTG | AATTCAAAAAGCAGATGAAGGCACGGTCACGCTCGAGGCAGATGAAGGCACGGTCACGG |
| Mimics NC | UUCUCCGAACGUGUCACGUTT | ACGUGACACGUUCGGAGAATT |
| miR-1297 mimics | UUCAAGUAAUUCAGGUG | CCUGAAUUACUUGAAUU |
| Inhibitor NC | CAGUACUUUUGUGUAGUACAA |  |
| MiR-1297 Inhibitor | CACCUGAAUUACUUGAA |  |
