# Supplementary figures and images for: miR-1297 sensitizes glioma cells to temozolomide (TMZ) treatment through targeting adrenomedullin (ADM)
Source: J Transl Med. 2022 Oct 1;20:443. doi: 10.1186/s12967-022-03647-6 (PMC9526964; doi:10.1186/s12967-022-03647-6)

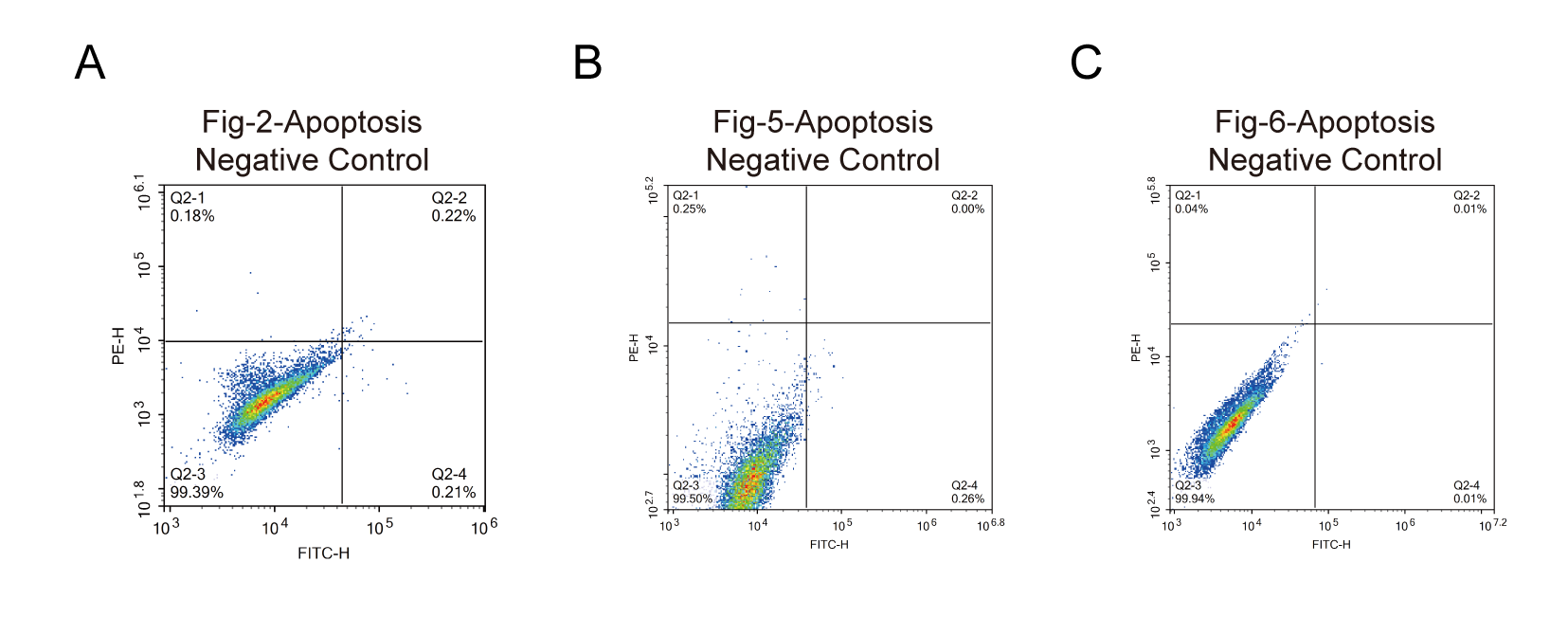

Supplement: Supplementary file 2 — Additional file 2: Fig. S1. The negative control of flow cytometry analysis. [file 12967_2022_3647_MOESM2_ESM.tif]

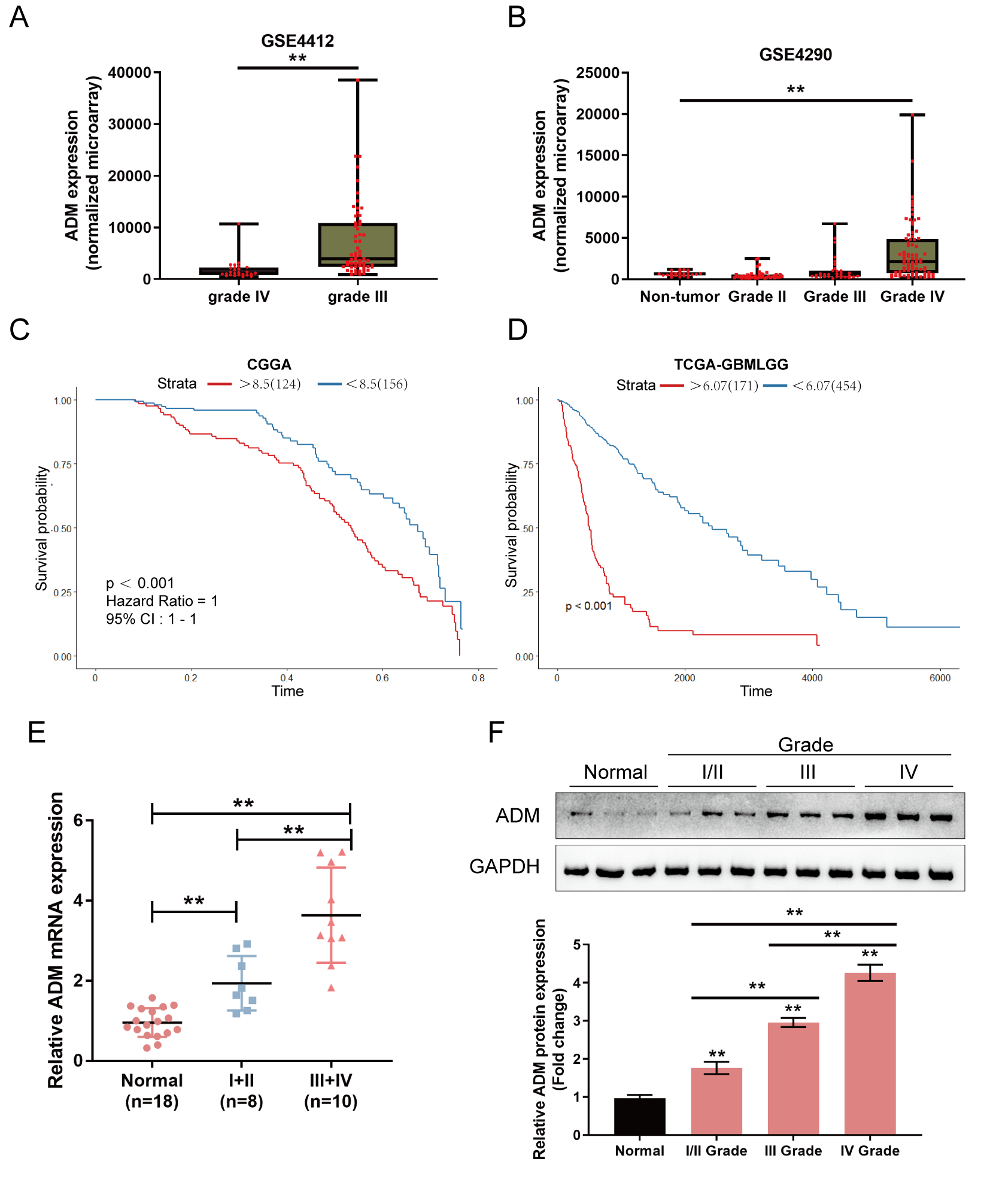

Supplement: Supplementary file 3 — Additional file 3: Fig. S2. ADM was upregulated in glioma tissues based on online datasets and clinical collected samples. (A) GSE4412; (B) GSE4290; (C) overall survival of ADM according to CGGA dataset; (D) overall survival of ADM according to TCGA-GBMLGG dataset; (E and F) The mRNA and protein levels of ADM in glioma samples and non-cancerous peritumoral brain edema tissue. [file 12967_2022_3647_MOESM3_ESM.tif]
